# Effectiveness of Knee Injury and Anterior Cruciate Ligament Tear Prevention Programs: A Meta-Analysis

**DOI:** 10.1371/journal.pone.0144063

**Published:** 2015-12-04

**Authors:** Laurel A. Donnell-Fink, Kristina Klara, Jamie E. Collins, Heidi Y. Yang, Melissa G. Goczalk, Jeffrey N. Katz, Elena Losina

**Affiliations:** 1 Orthopaedic and Arthritis Center for Outcomes Research, Department of Orthopedic Surgery, Brigham and Women's Hospital, Boston, Massachusetts, United States of America; 2 Harvard Medical School, Boston, Massachusetts, United States of America; 3 Division of Rheumatology, Immunology and Allergy, Brigham and Women’s Hospital, Boston, Massachusetts, United States of America; 4 Department of Biostatistics, Boston University School of Public Health, Boston, Massachusetts, United States of America; Mayo Clinic Minnesota, UNITED STATES

## Abstract

**Objective:**

Individuals frequently involved in jumping, pivoting or cutting are at increased risk of knee injury, including anterior cruciate ligament (ACL) tears. We sought to use meta-analytic techniques to establish whether neuromuscular and proprioceptive training is efficacious in preventing knee and ACL injury and to identify factors related to greater efficacy of such programs.

**Methods:**

We performed a systematic literature search of studies published in English between 1996 and 2014. Intervention efficacy was ascertained from incidence rate ratios (IRRs) weighted by their precision (1/variance) using a random effects model. Separate analyses were performed for knee and ACL injury. We examined whether year of publication, study quality, or specific components of the intervention were associated with efficacy of the intervention in a meta-regression analysis.

**Results:**

Twenty-four studies met the inclusion criteria and were used in the meta-analysis. The mean study sample was 1,093 subjects. Twenty studies reported data on knee injury in general terms and 16 on ACL injury. Maximum Jadad score was 3 (on a 0–5 scale). The summary incidence rate ratio was estimated at 0.731 (95% CI: 0.614, 0.871) for knee injury and 0.493 (95% CI: 0.285, 0.854) for ACL injury, indicating a protective effect of intervention. Meta-regression analysis did not identify specific intervention components associated with greater efficacy but established that later year of publication was associated with more conservative estimates of intervention efficacy.

**Conclusion:**

The current meta-analysis provides evidence that neuromuscular and proprioceptive training reduces knee injury in general and ACL injury in particular. Later publication date was associated with higher quality studies and more conservative efficacy estimates. As study quality was generally low, these data suggest that higher quality studies should be implemented to confirm the preventive efficacy of such programs.

## Introduction

Approximately seven million high school students participate in team sports each year [1] with 3–11% advancing to compete in NCAA college athletics [2]. Injuries occur frequently among these young athletes, with knee injuries accounting for 10–25% of all sports-related injuries [3]. Athletes involved in jumping, pivoting, or cutting, such as skiers or soccer players, are at increased risk for serious knee injuries including anterior cruciate ligament (ACL) tears. An estimated 250,000 ACL-related injuries occur annually in the United States [4], leading to 80,000 to 100,000 surgical ACL reconstruction surgeries per year [5]. Additionally, female athletes are 2 to 8 times more likely to injure their ACL compared to their male counterparts [6–8]. Serious knee injury may result in instability, damage to menisci or cartilage, reconstructive surgery and early osteoarthritis [9–11].

A growing number of prevention programs have been designed to reduce the incidence of knee injury in athletes, with many targeting ACL injuries specifically. These programs emphasize neuromuscular and proprioceptive training to reduce landing forces and adduction and abduction moments [12, 13]. Incorporated into these interventions are stretching, strengthening, and balance exercises as well as exercises that promote awareness of high-risk positions, enhance sports-specific agility, and improve technique. In four previously reported meta-analyses, injury prevention training programs significantly reduced knee and ACL injuries among young athletes [13–16]. However, these meta-analyses were limited by the number of studies they included and by the statistical methods utilized [13–16]. Our study adds substantially to the literature by almost doubling the numbers of ACL-specific studies included, by analyzing both knee and ACL injuries and by applying robust statistical methods. Our approach led to a more robust estimate of the association between injury prevention and neuromuscular/ proprioceptive intervention.

## Methods

This study was conducted according to the PRISMA (Preferred Reporting Items for Systematic Reviews and Meta-Analyses) protocol [17].

### Search Method

We performed a systematic literature search in the PubMed, MEDLINE/ EMBASE, CINAHL, Cochrane Central Register of Controlled Trials, and Web of Science databases through December 23, 2014. Our literature search was performed using the following search terms: [knee injury OR knee injuries OR anterior cruciate ligament injury OR anterior cruciate ligament injuries OR ACL injury OR ACL injuries OR lower limb injury OR lower limb injuries] AND [prevention]. We limited each search to peer-reviewed manuscripts published in English.

### Inclusion and Exclusion Criteria

Duplicate titles and studies published prior to 1996 were excluded following the literature search. Only literature published between 1996 and 2014 were included in order to capture the most recent trends in neuromuscular/proprioceptive prevention programs. Two reviewers (KK and MGG) independently screened unique studies based on the title and abstract and excluded studies that did not meet the selection criteria. Studies were considered for inclusion if the intervention used neuromuscular or proprioceptive training to prevent knee or ACL injuries in human subjects, and if the study outcomes included knee or ACL injury incidence. Review papers, editorials, lectures, commentaries, abstracts, trial design papers, case studies, surgical techniques, articles that were not peer-reviewed, and theses were excluded. Following the title/ abstract screen, MGG and KK independently reviewed the full text of those articles selected for inclusion to confirm that the studies met all inclusion criteria. When the two reviewers did not agree, a third reviewer (HYY) was consulted to reach a consensus. Following full paper review, KK and MGG examined the references of included studies to identify other relevant papers for analysis.

### Data Abstraction

Two reviewers (KK and MGG) independently abstracted the following data from all articles meeting inclusion criteria: first author, year of publication, title, sport type, subject sex, subject age, country in which the study was conducted, number of subjects in the control and intervention groups, intervention characteristics/ components, and knee and/or ACL injury outcome data. Reviewers scored each study based on the Jadad scale in order to measure the quality of included papers [18]. Abstracted data were compared, and discrepancies were adjudicated by a third author (HYY).

### Analysis

We used the incidence rate ratio (IRR) as the effect measure estimate, as it takes into consideration the variability in exposure time (exercise and play) among teams. The IRRs were obtained from each study or calculated from the number of injuries and exposure time if not provided. IRRs were combined into a weighted average, weighted by the precision of each IRR estimate (1/variance). In the case of clustered designs, variance estimates were conservatively adjusted for within team correlation [19].

We made a number of assumptions in our study. For trials that used a cluster design, when the intraclass correlation coefficient (ICC) was not reported, we assumed an ICC of 0.035 (mean ICC among those studies reporting ICC) to account for clustering outcomes within cluster groups, such as teams and coaches. Sensitivity analyses were conducted to test this assumption. Three studies did not report knee- or ACL-specific ICCs but reported ICCs for overall injuries or other lower limb injuries [11, 20, 21]. For these studies, we used reported ICCs as proxies for knee and ACL ICCs. Additionally, some studies performed interventions in multiple seasons [22, 23]. For these studies, we selected data from the first season to reduce the occurrence of repeat players and estimation bias (depletion of players more susceptible to injury) arising from one season to the next. A few studies did not report exposure (play and exercise) time [21, 24–27]. For these studies we assumed equal exposure time across treatment and control groups. Jadad scores were calculated to assess the methodological quality of each study (range 0 to 5; 5 indicating a rigorous study) [18].

We assessed publication bias graphically using funnel plots, and then assessed the between-study heterogeneity, first using funnel plots, and then with quantitative measures of heterogeneity, including statistical influence, inconsistency, and other measures (H, I^2^ and Q-term). Per convention, negative values of I^2^ were set to zero [28]. These measures were factored into decisions to retain or exclude specific studies from the analysis [29]. Studies with a strong influence on heterogeneity were excluded from our main analysis, though we included all studies in a sensitivity analysis. Meta-analysis summary estimates were based on the study IRRs weighted by their precision using a random effects model. We used forest plots summarizing the natural log of the IRR across studies to depict results of the meta-analysis graphically. The vertical line at ln IRR = 0 provides a reference for a null result. We used the ln IRR so that the confidence intervals are symmetrical about the means and to accurately display IRRs that are less than one.

We used meta-regression to determine the effect of various training strategies and study characteristics, including the year of publication, on the incidence rate ratio [30]. We examined the following technical components: balance training, plyometric (jump) training, strength/resistance training, running technique training (combined technique training and running exercises (e.g. shuttle run, bounding run, etc.)), and stretching. We created a composite score to evaluate whether programs with more components had better or worse outcomes by summing the number of technical components (possible range: 0 to 5). We also examined age of the cohort (high school or younger vs. older than high school) and whether the intervention included pre-season training. Finally, we conducted a subgroup analysis restricted to studies that reported non-contact injuries in order to identify the efficacy of intervention on non-contact ACL injuries.

## Results

### Studies Included in the Analysis

**Fig 1:**
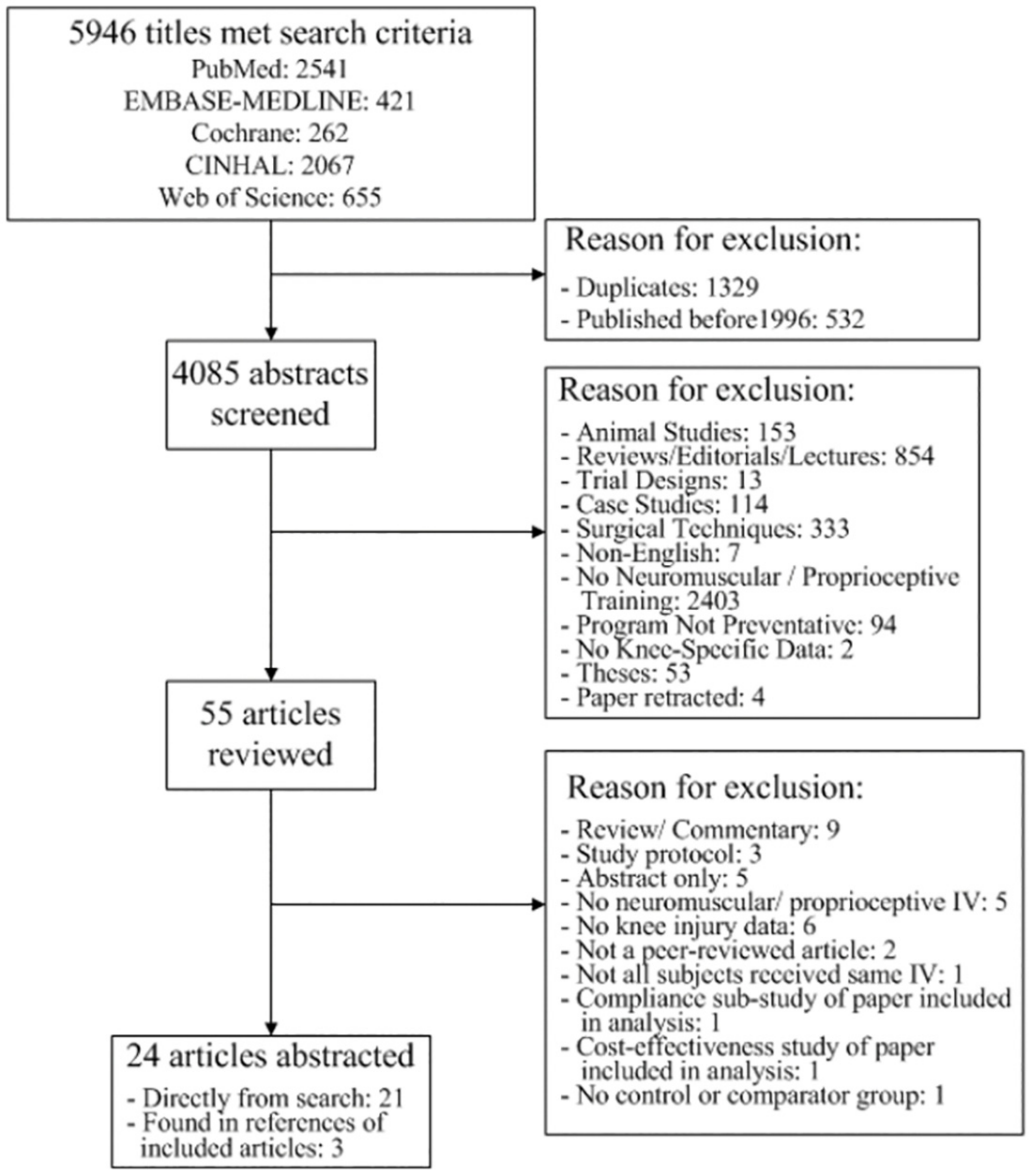
PRISMA Flow Diagram.

The initial search algorithm returned 5,946 titles. Fig 1 presents the literature review search results. Twenty-four studies met our inclusion criteria and were therefore analyzed to evaluate the effect of neuromuscular or proprioceptive training on knee and ACL injury prevention. Of the 24 studies, 1 took place in Australia [21], 1 in Canada [31], 7 in the United States [22, 24, 32–36], and the remaining 15 took place in Europe (Denmark [37], Finland [20], Switzerland [38], Germany [26], Greece [27], Italy [25, 39], Netherlands [40], Norway [11, 23, 41, 42], or Sweden [43–45]). Fourteen of the interventions were carried out on soccer players, 4 on handball players, 1 on floorball players, 1 on basketball players and 1 on Australian Army recruits. Three studies intervened on multiple sports (2 studies focused on soccer, basketball and volleyball, and 1 study focused on soccer and basketball). The mean study sample was 1,093 subjects (standard deviation [SD] 1,077). Fifteen of the studies focused on women only; four focused on men only; three included men and women, and two studies did not report the sex of study subjects. Five studies used a Federation International de Football Association (FIFA) training program [36, 38–40, 42], 3 studies used a Prevent Injury and Enhance Performance (PEP) or modified PEP program [22, 34, 41], 1 study used the Frappier Acceleration Training Program [24], 1 study used the HarmoKnee Preventive Training Program [44], 1 study used the plyometric-based knee ligament injury prevention (KLIP) program [33], and 13 studies used proprietary programs. Sixteen studies reported data on ACL injury; however, in 2 of these studies, one or both groups experienced zero ACL injuries [36, 44]. As a result, the IRR could not be calculated for these studies, and they were not included in the ACL meta-analysis. A sensitivity analysis was conducted to include these studies after assigning a value of 0.5 to zero injury counts. Twenty studies reported data on knee injury, seventeen of which included all knee injury types and three of which [20, 27, 32] defined knee injuries specifically as knee ligament injuries. Seven studies reported both contact and non-contact injuries [20, 23, 26, 32, 34, 44, 45], while 4 studies reported non-contact injuries only [21, 22, 33, 35]. Thirteen studies did not specify whether knee/ ACL injuries were contact or non-contact.

### Knee Injury Prevention

**Fig 2:**
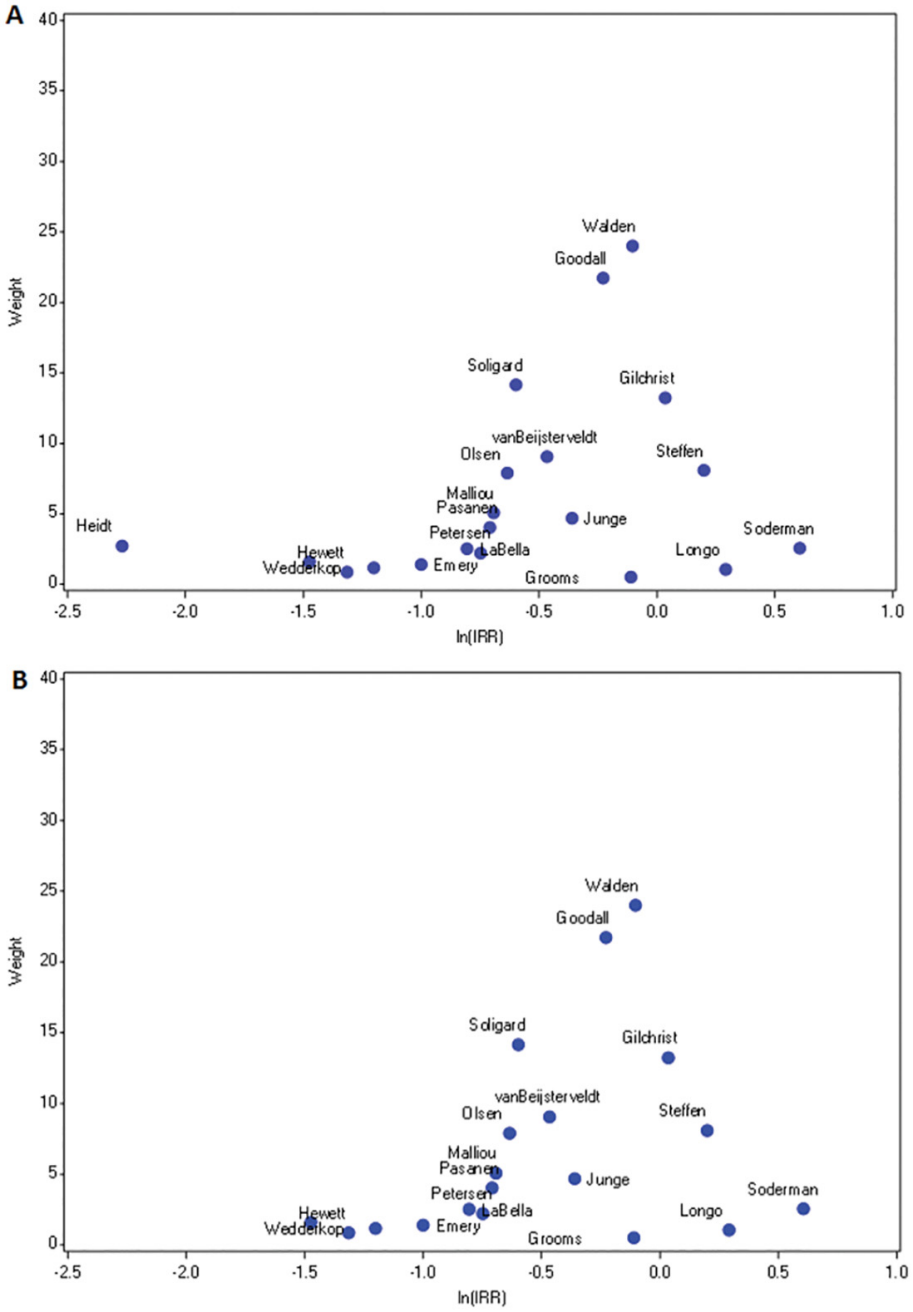
Sensitivity Analyses: Funnel plots of weight by natural log of the incidence rate ratio for knee injury. Panel A includes all 20 studies of knee injury, while Panel B includes only 19 studies of knee injury (excluding Heidt et al).

Twenty (of 24) studies evaluated prevention of knee injury [11, 20, 21, 24, 26, 27, 31, 32, 34–45]. The second to last column of Table 1 lists the IRR and 95% confidence intervals for each of these studies. Fig 2 displays funnel plots of precision (weight) by natural log of the IRR. The plot for knee injury has two peaks (Fig 2A), indicating potential heterogeneity. The plot also shows some skewness with more studies falling toward the left tail (indicating superiority of the intervention). Quantitative measures indicate moderate inconsistency and heterogeneity (I^2^ = 0.294, H = 1.190 respectively). The estimates from Heidt et al (depicted on the plot) contributed the most substantial weight to the heterogeneity score (Q-term = 9.965) and had high influence (Influence = 0.215). After eliminating Heidt et al from the analysis, the 19 remaining studies were depicted by a funnel plot with a single peak (Fig 2B). The plot shows symmetry around the peak, failing to suggest publication bias. Quantitative measures indicate low heterogeneity (I^2^ = 0, H = 0.964 respectively), which support combining individual studies (excluding Heidt et al) into a single summary estimate.

**Table 1:** Study specific incident rate ratio (95% confidence interval) for the impact of neuromuscular training programs to reduce knee or anterior cruciate ligament (ACL) injury.

									Incidence Rate Ratio (95% Confidence Interval)	
	First Author (Date)	Study Design	Sample Size	Sport	Jadad Score	Sex	Age (High School-aged vs. Older than High School)	Program Components^1^	Knee Injury	ACL Injury
1	Goodall[21] (2013)	Cluster randomized trial	779	Military Training	3	Female, Male	Older than High School	P, B, R/T	0.796 (0.523, 1.212) †	
2	Grooms[36] (2013)	Prospective cohort study	64	Soccer	1	Male	Older than High School	P, B, S/R, R/T, S	0.895 (0.056, 14.303) ‡	
3	vanBeijsterveldt[40] (2012)	Cluster randomized trial	456	Soccer	1	Male	Older than High School	P, B, S/R, R/T, S	0.627 (0.327, 1.203) ‡	
4	Walden[45] (2012)	Cluster randomized trial	4,564	Soccer	3	Female	High School-aged	P, B, S/R, R/T	0.902 (0.604, 1.346)	0.433 (0.175, 1.072)
5	Longo[39] (2012)	Cluster randomized trial	121	Basketball	1	Male	High School-aged	P, B, S/R, R/T, S	1.338 (0.199, 8.991) ‡	
6	LaBella[35] (2011)	Cluster randomized trial	1,492	Soccer Basketball	3	Female	High School-aged	P, S/R, R/T	0.446 (0.130, 1.537) ‡	0.164 (0.025, 1.080) ‡
7	Emery[31] (2010)	Cluster randomized trial	744	Soccer	1	Female, Male	High School-aged	P, B, S/R, R/T, S	0.368 (0.070, 1.940)	
8	Kiani[44] (2010)	Prospective cohort study	1,506	Soccer	0	Female	High School-aged	P, B, S/R, R/T	0.229 (0.049, 1.071) ‡	
9	Soligard[41] (2008)	Cluster randomized trial	1,892	Soccer	1	Female	High School-aged	P, B, S/R, R/T, S	0.549 (0.326, 0.925)	
10	Gilchrist[34] (2008)	Cluster randomized trial	1,435	Soccer	1	Female	Older than High School	P, S/R, R/T, S	1.036 (0.605, 1.776) ‡	0.584 (0.182, 1.878) ‡
11	Pasanen[20] (2008)	Cluster randomized trial	457	Floorball	3	Female	Older than High School	P, B, S/R, S, R/T	0.493 (0.186, 1.307)	1.161 (0.315, 4.274)
12	Steffen[42] (2008)	Cluster randomized trial	2,020	Soccer	3	Female	High School-aged	P, B, S/R, R/T, S	1.220 (0.612, 2.433)	0.792 (0.120, 5.205)
13	Pfeiffer[33] (2006)	Prospective cohort study	1,439	Soccer, Basketball, Volleyball	0	Female	High School-aged	P, R/T		2.153 (0.321, 14.447) ‡
14	Mandelbaum[22] (2005)	Prospective cohort study	2,946*	Soccer	0	Female	High School-aged	P, S/R, R/T, S		0.114 (0.018, 0.723) ‡
15	Petersen[26] (2005)	Prospective matched cohort	276	Handball	1	Female	Older than High School	P, B, R/T	0.474 (0.127, 1.765)† ‡	0.190 (0.014, 2.523) † ‡
16	Olsen[11] (2005)	Cluster randomized trial	1,837	Handball	2	Female, Male	High School-aged	P, B, S/R, R/T	0.530 (0.264, 1.064)	0.280 (0.045, 1.747)
17	Malliou[27] (2004)	Prospective cohort study	100	Soccer	0	Not Reported	High School-aged	B	0.500 (0.209, 1.194) † ‡	
18	Myklebust[23] (2003)	Prospective cross-over study	1,797*	Handball	0	Female	Older than High School	P, B, R/T		0.960 (0.491, 1.875) ‡
19	Junge[38] (2002)	Prospective cohort study	194	Soccer	1	Male	High School-aged	P, B, S/R, R/T, S	0.697 (0.283, 1.721) ‡	
20	Heidt[24] (2000)	Randomized trial	300	Soccer	1	Female	High School-aged	P, S/R, R/T	0.103 (0.032, 0.340)† ‡	0.125 (0.016, 0.999)† ‡
21	Soderman[43] (2000)	Cluster randomized trial	140	Soccer	2	Female	Older than High School	B	1.831 (0.537, 6.240) ‡	5.492 (0.434, 69.533)‡
22	Hewett[32] (1999)	Prospective cohort study	829	Soccer, Volleyball, Basketball	0	Female	High School-aged	P, S/R, R/T, S	0.269 (0.033, 2.217) ‡	0.537 (0.055, 5.251) ‡
23	Wedderkop[37] (1999)	Cluster randomized trial	237	Handball	1	Female	High School-aged	P, B, S/R	0.301 (0.050, 1.812) ‡	
24	Caraffa[25] (1996)	Prospective cohort study	600	Soccer	0	Not Reported	Older than High School	B		0.143 (0.064, 0.321) † ‡

^1^ P: plyometric (jump training); B: balance exercises; S/R: strength/ resistance training; R/T: running/ technique training exercises (e.g. shuttle run, bounding run, etc.); S: stretching

^2^ Average age reported for injured players only

^†^ No estimate of exposure time.

IRR estimates were calculated assuming equal exposure time across groups.

^‡^ No correlation coefficient or inflation factor reported.

Confidence intervals were calculated assuming a correlation coefficient of 0.035

* Only control season and first intervention season included

**Fig 3:**
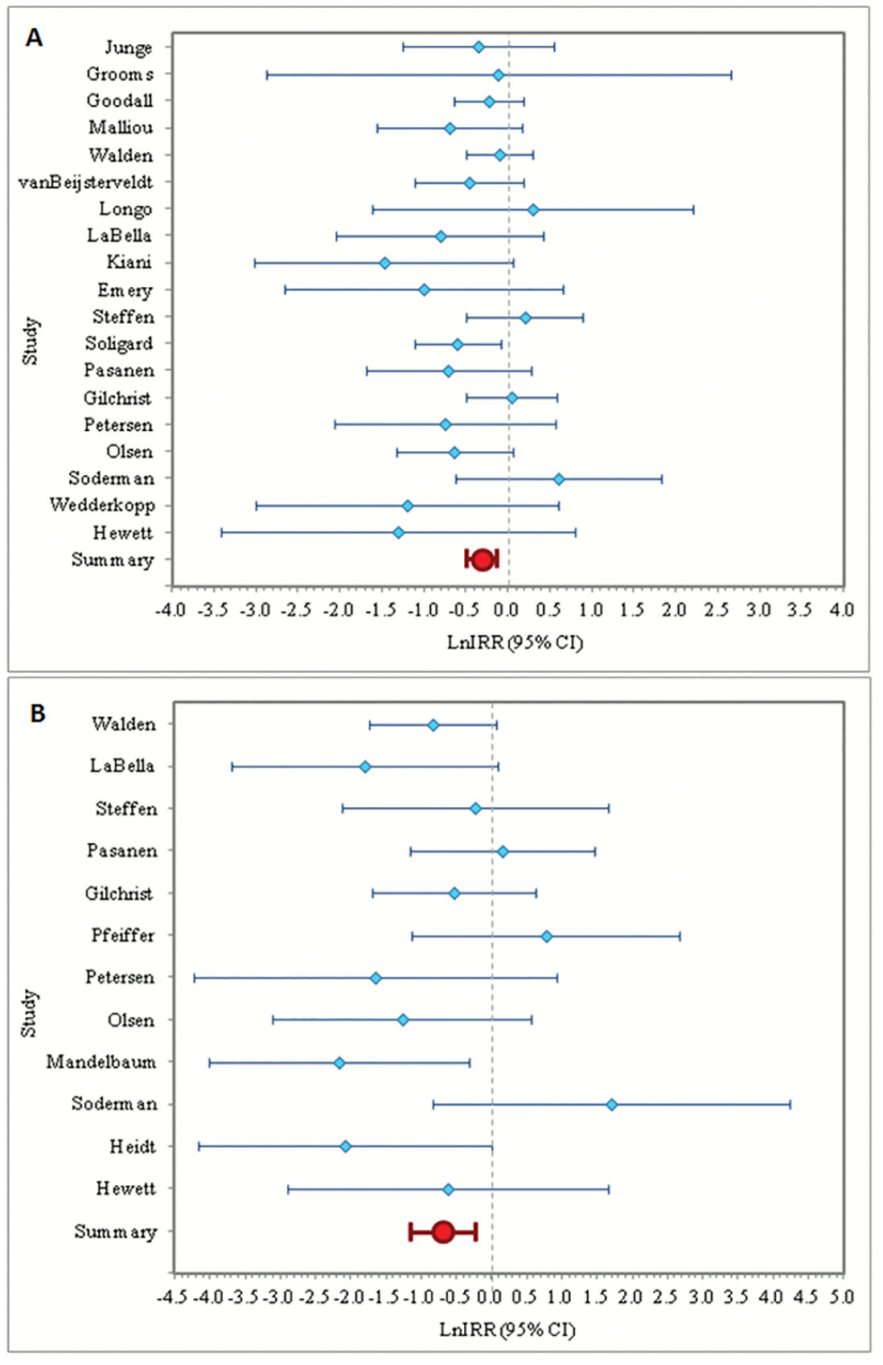
Forest plots of the natural log of IRR and 95% confidence interval for knee and ACL injuries excluding studies that contribute to heterogeneity. Summary estimates from the meta-analysis are presented at the bottom of the plot in red. A) Forest plot of the natural log of IRR and 95% confidence interval for knee injury excluding Heidt et al. B) Forest plot of the ln IRR and 95% confidence interval for ACL injury excluding Caraffa et al and Myklebust et al.

The meta-analysis random-effect IRR (excluding Heidt et al) was 0.731 (95% CI: 0.614, 0.871), indicating that neuromuscular/ proprioceptive interventions significantly reduced knee injury by 26.9%. The results of the meta-analysis for knee injury prevention are presented graphically in a forest plot (Fig 3A).

### ACL Injury Prevention

**Fig 4:**
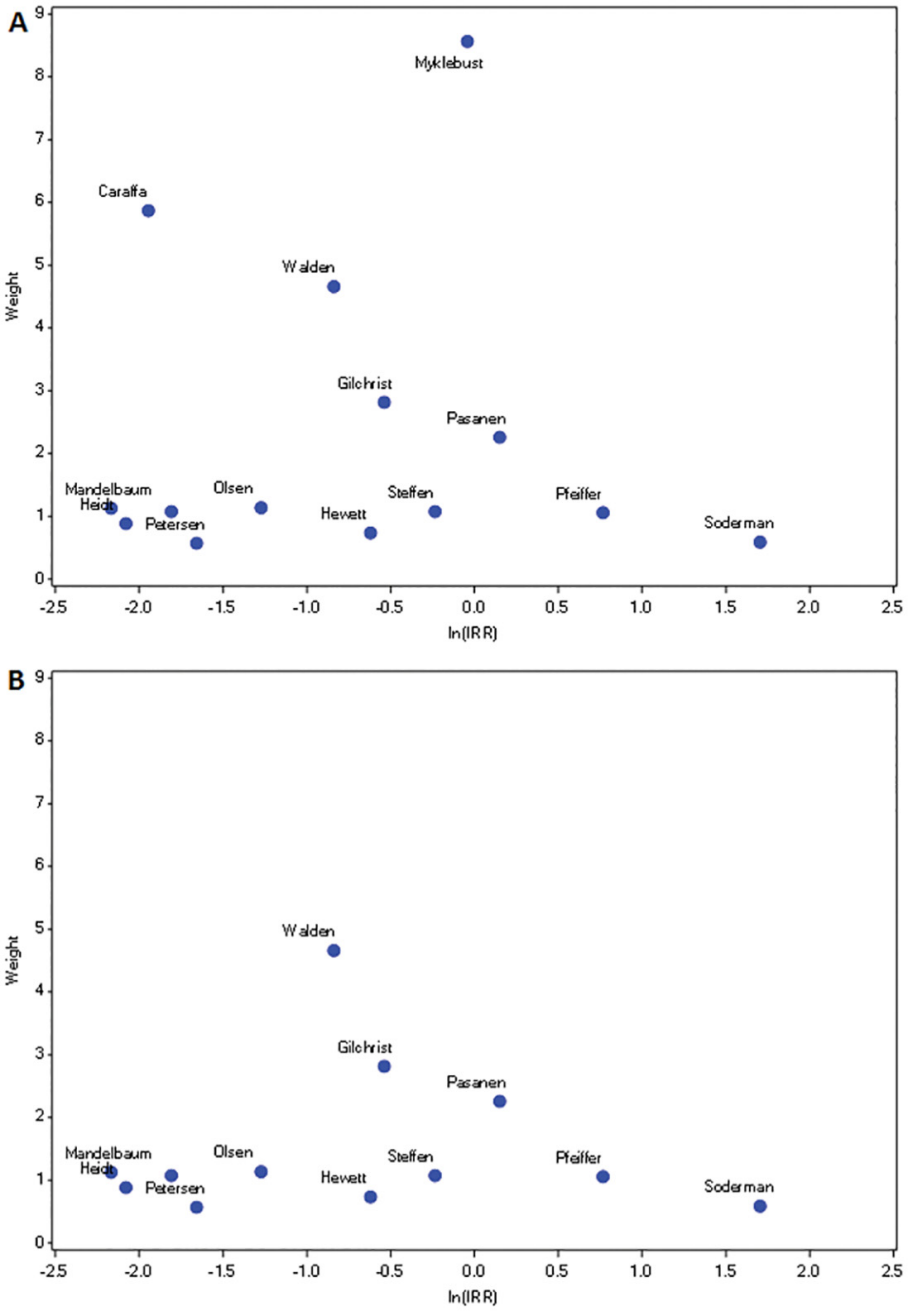
Sensitivity Analyses: Funnel plots of weight by natural log of the incidence rate ratio for ACL injury. Panel A includes all 14 studies of ACL injury, while Panel B includes only 12 ACL studies (excluding Caraffa et al and Myklebust et al).

Sixteen studies evaluated prevention of ACL injury [11, 20, 22–26, 32–35, 42, 43, 45]. The last column of Table 1 lists the IRR and 95% confidence intervals for impact of the program on ACL injury prevention. A funnel plot of the 14 ACL studies analyzed (Fig 4A) is relatively symmetric, but depicts two peaks, indicating potential heterogeneity. Quantitative measures of heterogeneity also estimated moderate inconsistency and heterogeneity (I^2^ = 0.516, H = 1.438 respectively). The estimates from Myklebust et al [23] and Caraffa et al [25] (depicted on the plot) were the most influential (Influence = 1.547, 1.848 respectively). These studies also contributed substantial weight to the heterogeneity score (Q-term = 4.322, 8.374 respectively). Soderman et al [43] had high heterogeneity score (Q-term = 3.59), but was not influential (Influence = 0.067). Therefore we decided to retain the study by Soderman et al in our analysis. After eliminating studies by Caraffa et al and Myklebust et al, the 12 remaining studies displayed a more balanced distribution in the funnel plot with a single peak (Fig 4B). Further, quantitative measures of heterogeneity dropped well below moderate levels (I^2^ = 0.221; H = 1.133).

The meta-analysis random-effect IRR (excluding Caraffa et al and Myklebust et al) was 0.493 (95% CI: 0.285, 0.854), indicating that neuromuscular/proprioceptive interventions significantly reduced ACL injury by 50.7%. The results of the meta-analysis for ACL injury prevention are presented graphically in a forest plot in Fig 3B. These results do not include two studies that reported zero ACL injuries in one or both groups [36, 44].

### Meta-Regression

Among knee injury studies, none of the specific training components were statistically significantly associated with outcome in meta-regression (Table 2). Two studies included 1 of 5 training components (Malliou and Soderman, balance training only), 5 studies included 3 components, 5 studies included 4 components, and 8 studies included all 5 technical components. We did not find an association between number of components and outcome when evaluating the technical components (p = 0.5448), and there were no obvious trends (e.g., more components being associated with better outcomes or vice versa). We also did not find a statistically significant association between training components and outcome among ACL injury studies. Again, none of the composite measures were significantly associated with outcome.

**Table 2:** Results of Meta-Regression.

	Knee Injury			ACL Injury		
Component	n (%)	IRR	P-value*	n (%)	IRR	P-value*
Balance training			0.3677			0.5142
No	4 (20%)	0.503		6 (43%)	0.359	
Yes	16 (80%)	0.681		8 (57%)	0.530	
Plyometric (jump) training			0.5907			0.5182
No	2 (10%)	0.810		2 (14%)	0.497	
Yes	18 (90%)	0.639		12 (86%)	0.311	
Strength/ resistance Training			0.5268			0.4567
No	4 (20%)	0.751		5 (36%)	0.389	
Yes	16 (80%)	0.624		9 (64%)	0.608	
Running Technique training			0.8871			0.5182
No	3 (15%)	0.690		2 (14%)	0.497	
Yes	17 (85%)	0.652		12 (86%)	0.311	
Stretching			0.4007			0.6638
No	10 (50%)	0.587		9 (64%)	0.547	
Yes	10 (50%)	0.723		5 (36%)	0.421	
Age			0.1995			0.4097
High School	13 (65%)	0.791		8 (57%)	0.363	
> High School	7 (35%)	0.579		6 (43%)	0.581	
Intervention Period			0.0016			0.3281
Pre-Season	5 (25%)	0.237		5 (36%)	0.323	
During Season only	15 (75%)	0.754		9 (64%)	0.573	

* The p-value tests a difference in IRR between categories.

Age, classified as high school aged or younger versus older than high school aged, was not significantly associated with outcome for either knee or ACL injuries. Having training as part of the pre-season (pre-season only or pre-season and in-season) versus in-season only was associated with a lower risk of knee injury (p = 0.0016). The trend for a lower risk of injury was also evident for ACL injuries, though this did not reach statistical significance (p = 0.3281).

**Fig 5:**
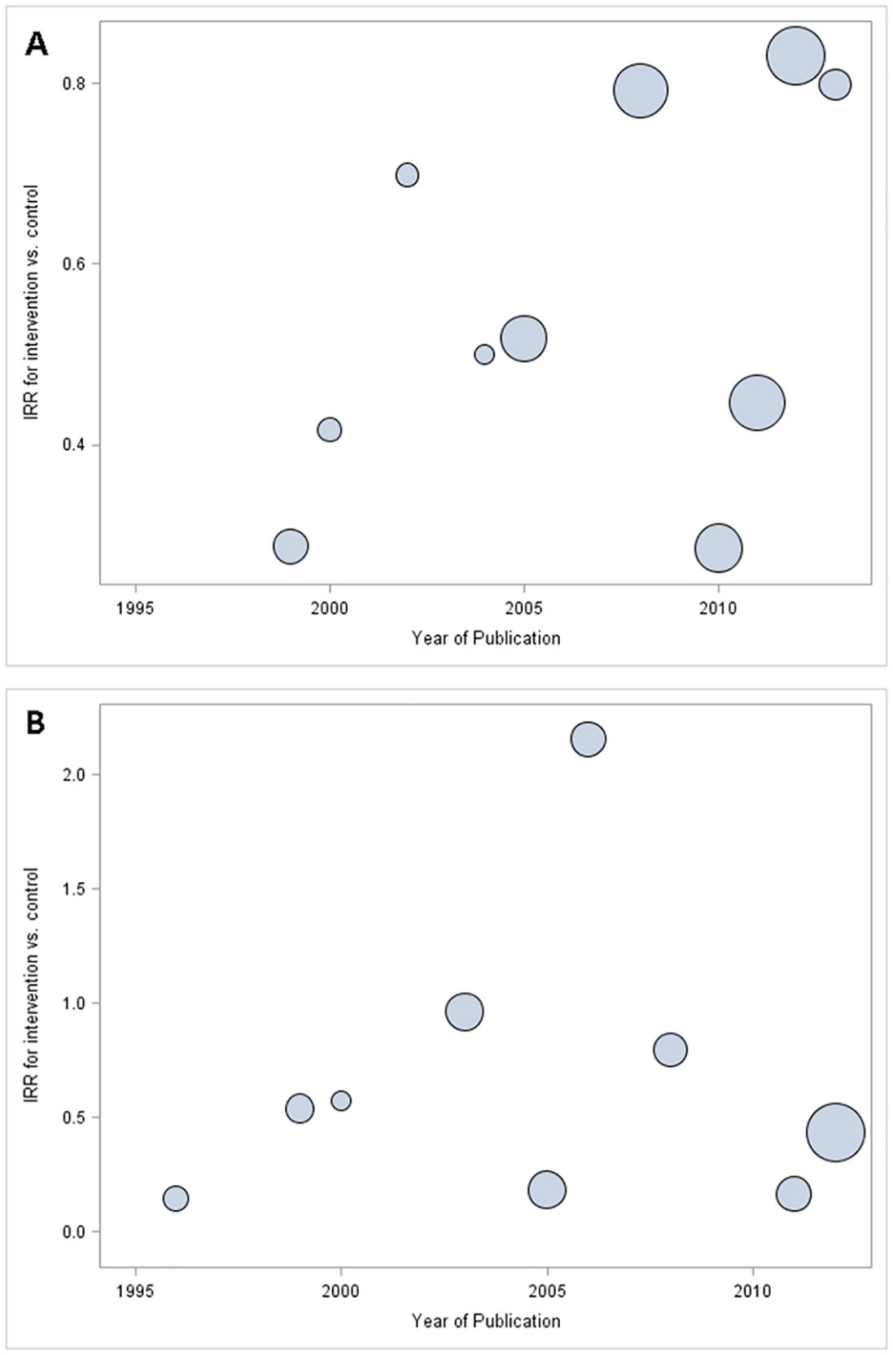
Meta-Regression: Year of Publication. This figure shows the association between the year of publication and intervention efficacy for A) knee injury and B) ACL injury. Publication year is along the X-axis, and each dot represents the summary IRR for that year. The size of the bubble corresponds to the average sample size for studies published in that year.

Later year of publication was associated with more conservative estimates of intervention efficacy. For knee injury the p-value for the trend was 0.0544. For ACL injury the association had less certainty (p = 0.3417) (Fig 5). Higher Jadad scores were associated with more conservative estimates of intervention efficacy. The trend reached statistical significance for knee injury (0.0289) and did not for ACL injury (0.5913).

### Subgroup Analysis

We performed a subgroup analysis to assess the effectiveness of prevention intervention on non-contact injuries. Nine studies reported non-contact ACL injuries [20, 22, 23, 26, 32–35, 45]. Two studies were excluded because they reported injury counts of zero [36, 44]. The meta-analysis random-effect IRR for the 7 remaining studies was 0.513 (95% CI: 0.298, 0.884).

### Sensitivity Analyses

#### Knee Injury

**Fig 6:**
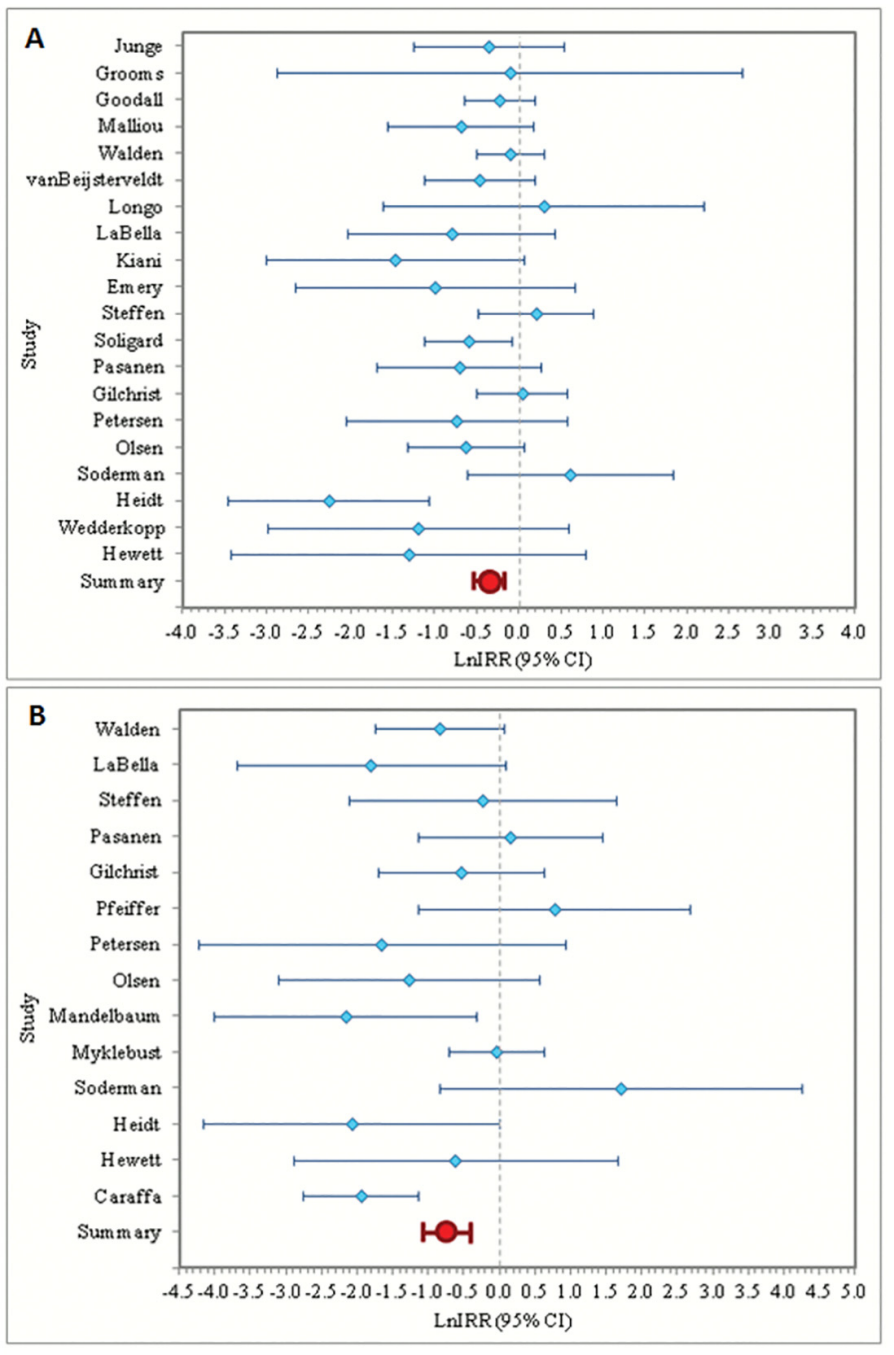
Sensitivity Analyses. Forest plots of the ln IRR and 95% confidence interval, including studies that contribute to heterogeneity. Panel A shows the forest plot for knee injury, including Heidt. Panel B shows the forest plot for ACL injury, including Caraffa and Myklebust. Summary estimates from the meta-analysis are presented at the bottom of the plot in red.

We assessed the effectiveness of intervention including studies with strong influence of heterogeneity. Results are presented graphically in a forest plot (Fig 6A). Inclusion of Heidt et al in the analysis of knee injury prevention changed the random-effect IRR of knee injury from 0.731 to 0.658 (95% CI: 0.523, 0.827). This result was consistent and indicated a significant reduction of risk of knee injury in neuromuscular/ proprioceptive intervention groups. Next, we evaluated the study assumption that ICC = 0.035 for studies where ICC was not reported. The maximum of reported ICC was 0.071. We tested a range of intraclass correlation coefficients between 0.000 and 0.080 and found that varying intraclass correlation coefficients did not affect the results.

#### ACL Injury

Inclusion of Caraffa et al and Myklebust et al in the analysis of ACL injury prevention resulted in a random-effect IRR of ACL injury of 0.460 (95% CI: 0.264, 0.804), close to the main analysis IRR of 0.493. These results are presented graphically in a forest plot (Fig 6B). Additionally, two studies (Kiana et al and Grooms et al) reported zero ACL injuries in one or both groups. We conducted a sensitivity analysis including these two studies with a 0.5 correction for zero injury counts. The results remained consistent (random-effect IRR = 0.466 [95% CI: 0.331, 0.656]). As with the knee injury analysis, we evaluated the study assumption that ICC = 0.035 for studies where ICC was not reported and found that varying the ICC did not affect the results.

## Discussion

In the current study, we aimed to summarize the effects of neuromuscular and proprioceptive training on knee and ACL injury reduction. We conducted a meta-analysis of 24 controlled trials of preventive interventions for knee and ACL injuries. Using an overall IRR estimate as the summary estimate of effect, both the studies of knee injury and the studies of ACL injury demonstrated statistically significant reductions in injury rates associated with preventive interventions. We found that neuromuscular and proprioceptive prevention programs appeared to reduce knee injuries by 26.9% and ACL injuries by 50.7%.

Among the 20 studies reporting knee injury rates, four [32, 35, 40, 44] reported a statistically significant association between the intervention and knee injury prevention in their original manuscripts. Twelve studies reported a reduction in knee injuries that did not reach statistical significance. Nine studies reported a significant reduction in total injuries examined [11, 20, 24, 27, 36–39, 41]. Our primary meta-analysis of IRR estimates supported the protective effect of neuromuscular and proprioceptive training on knee injury reduction.

Among the 14 studies reporting ACL injury rates, 4 reported a statistically significant association between the intervention and injury prevention in their original manuscripts [22, 25, 32, 45]. Seven studies reported a reduction in knee injuries that did not reach statistical significance. Our primary meta-analysis of IRR estimates supported this protective finding.

Our findings build upon several previous meta-analyses evaluating ACL prevention methods conducted by Hewett et al, Yoo et al, Grimm et al and Sadoghi et al [13–16]. All analyses conducted by Hewett et al (2006), Yoo et al (2010) and Sadoghi el al (2012) found a significant protective effect of prevention programs on ACL injuries. The magnitude of the effect was similar for the three studies: Hewett et al included 6 studies and found an odds ratio of 0.40 (95% CI: 0.26, 0.61); Yoo et al analyzed seven studies (including all 6 of those in Hewett et al) and found an odds ratio of 0.40 (95% CI: 0.27, 0.60); Sadoghi et al included 8 studies (5 included by Hewett et al or Yoo et al) and found a risk ratio of 0.38 (95% CI: 0.20, 0.72). All three analyses were limited to females only and did not assess the effect of prevention programs on the more general grouping of knee injuries. In 2014, Grimm et al conducted a meta-analysis to assess the protective effects of knee injury prevention programs on knee and ACL injury incidence among male and female athletes. They limited their study to Level I randomized controlled trials of soccer players. Their analysis included nine studies, seven of which were not included in any of the previous meta-analyses [43]. They observed a statistically significant reduction in the risk of knee injury, with a summary risk ratio of 0.74 (95% CI: 0.55, 0.98). The prevention programs showed a protective effect for ACL injury, but this did not reach statistical significance, with a summary risk ratio of 0.66 (95% CI: [0.33, 1.32], p = 0.238).

Results of analyses examining specific training components have been mixed. Sadoghi et al did not find a statistically significant association between balance board use or use of video assistance and injury prevention [14], while Yoo et al found a protective but non-significant effect of plyometric and strengthening components in subgroup analysis [15]. In our analysis, we did not find a significant association between any single training component and injury prevention, neither for ACL injury nor for knee injury. We did find that interventions started in the pre-season (IRR 0.237), rather than during the season (IRR 0.754), were better at preventing knee injuries (p = 0.0016) and had a protective but non-significant effect for ACL injuries. Sadoghi et al also found a protective, non-significant, effect of pre-season interventions for ACL injuries [14]. These results suggest that it may not be the individual program components that are important, but the timing of the intervention.

Since Sadoghi’s meta-analysis, four additional ACL studies have been published [35, 36, 44, 45], only one of which [45] was included in Grimm et al. We have also added five older studies that met our inclusion criteria [11, 20, 23, 42, 43], only two of which [42, 43] were used in Grimm et al. In our analysis the IRR estimate was selected as the measure of effect rather than the odds ratio or risk ratio, as the IRR adjusts for exposure time. Variances were conservatively adjusted for within team correlation in clustered designs, and sensitivity analyses were conducted to test the assumptions of design effects. These methods were not employed by Hewett et al, Sadoghi et al, Yoo et al, or Grimm et al, although these meta-analyses included studies with clustered designs. Additionally, we addressed the limitations in the assessment of heterogeneity in previous meta-analyses [13, 16]. We assessed heterogeneity both graphically and quantitatively. Based on our assessment, we identified and excluded studies that contributed substantially to heterogeneity and selected the most appropriate meta-analytic modeling methods. We used multiple sensitivity analyses to confirm our primary findings.

The results of the meta-analysis reported in this paper should be viewed within the limitations of the included studies. The majority of the studies (63%) included in our analysis focused on injury prevention exclusively in female athletes; therefore, our results should be generalized cautiously to male athletes. Thirteen [11, 24, 25, 27, 31, 36–43] of the included studies did not distinguish between contact and noncontact knee or ACL injuries; therefore, in our main analysis, we analyzed all ACL injuries (contact and noncontact) when both were reported. In a subgroup analysis, we examined non-contact ACL injuries exclusively and found comparable results but were limited in the number of studies that we could include. The injury prevention programs reported in the studies included in the current meta-analysis used the same underlying principles of neuromuscular training but varied in the precise way in which these principles were implemented. For example, Gilchrist and colleagues used the Prevent Injury and Enhance Performance (PEP) Program while Pfeiffer and colleagues used the Knee Ligament Prevention (KLIP) Program [33, 34]. Both programs use proprioceptive and neuromuscular exercises, yet they differ in the specific drills used to accomplish the training (e.g. straight jumps compared to lateral hops over 2 to 6 inch cones). Other programs implemented their own individual training regimens and did not use an established program. This may have limited our ability to detect differences in effectiveness by training components in meta-regression. Data on compliance with the training programs were not consistently reported or readily available. Most papers (56%) analyzed and reported data only on those subjects who completed the study as opposed to all subjects who began the study. Finally, it is possible that injury prevention training has a greater impact on specific sports, such as soccer or handball, where more cutting and pivoting occur. More publications with sport-specific data are needed to evaluate the impact of such programs on sport-specific injury prevention.

We were able to confirm that neuromuscular and proprioceptive training has a protective effect on knee injury incidence, including ACL-specific knee injuries, in athletes. Our analyses showed a statistically significant 27% reduction in knee injury rate and 51% reduction in ACL injury rate specifically. We suggest that athletic departments and coaches consider implementation of neuromuscular and proprioceptive injury prevention programs as a part of regular training given their protective effect on knee injury incidence and the potential to reduce the burden of knee OA [10]. We also suggest that further research focus on elucidating the specific components of neuromuscular and proprioceptive training that contribute to the prevention of knee injury.

## Supporting Information

S1 PRISMA Checklist PRISMA Checklist. (PDF)
